# Added Value to GLP-1 Receptor Agonist: Intermittent Fasting and Lifestyle Modification to Improve Therapeutic Effects and Outcomes

**DOI:** 10.3390/biomedicines13123079

**Published:** 2025-12-13

**Authors:** Dragos Cozma, Cristina Văcărescu, Claudiu Stoicescu

**Affiliations:** 1 Institute of Cardiovascular Diseases Timisoara, 300310 Timisoara, Romania; dragos.cozma@umft.ro; 2 Cardiology Department, Victor Babeș University of Medicine and Pharmacy, 300041 Timisoara, Romania; 3 Research Center of the Institute of Cardiovascular Diseases Timisoara, 300310 Timisoara, Romania; 4 Cardiology and Cardiovascular Surgery Department, University of Medicine and Pharmacy Carol Davila, 37 Dionisie Lupu, 030167 Bucharest, Romania; claudiu.stoicescu@umfcd.ro; 5 Cardiology and Cardiovascular Surgery Department, University Emergency Hospital, 169 Splaiul Independenței, 050098 Bucharest, Romania

**Keywords:** GLP-1 receptor agonists, intermittent fasting, metabolic flexibility, obesity management, longevity

## Abstract

Obesity remains a major global health challenge, with glucagon-like peptide-1 receptor agonists (GLP-1RAs) providing substantial yet sensitive benefits in weight reduction, glycemic control, and cardiovascular protection. Despite robust trial data, real-world persistence is limited by cost, tolerability, and hedonic adaptation. Intermittent fasting and time-restricted eating offer physiologically complementary, low-cost strategies that enhance fat oxidation, insulin sensitivity, and metabolic flexibility while engaging behavioral mechanisms of self-control and dietary regularity. This narrative review synthesizes current evidence and proposes a pragmatic, phased framework integrating GLP-1RA therapy with structured intermittent fasting and protein-optimized nutrition. The model emphasizes sequential initiation, transition, and maintenance phases designed to align pharmacologic appetite suppression with lifestyle-driven metabolic remodeling. Mechanistically, GLP-1RAs target vascular and neuroendocrine pathways, whereas fasting activates nutrient-sensing networks (AMPK, mTOR, sirtuins) associated with autophagy and longevity. Combined application may preserve lean mass, improve psychological autonomy, and reduce healthcare costs. Future research should validate this hybrid strategy in randomized trials assessing long-term weight durability, functional outcomes, and cost-effectiveness. By uniting pharmacologic potency with behavioral sustainability, phased GLP-1–fasting integration may represent an effective, affordable, and longevity-oriented paradigm for metabolic health.

## 1. Introduction

Obesity remains among the most pressing global health challenges of the 21st century. Despite significant pharmacological advances, including the use of glucagon-like peptide-1 receptor agonists (GLP-1 RAs), long-term adherence and sustained weight loss remain limited for a considerable proportion of patients [1] Clinical trials have demonstrated that GLP-1 RAs achieve substantial improvements in weight reduction, glycemic control, and cardiovascular outcomes, yet real-world persistence often declines over time, driven by cost, side effects, and psychological adaptation to blunted food reward [2,3,4].

GLP-1RAs and dual incretin therapies have redefined medical management of obesity, delivering average weight loss in the ~10–20% range and—critically—cardiovascular risk reduction in high-risk populations. Semaglutide 2.4 mg (STEP-1) and tirzepatide (SURMOUNT-1) established the magnitude and durability of weight loss under trial conditions, while SELECT demonstrated semaglutide’s ability to lower major adverse cardiovascular events in adults with established atherosclerotic disease and no diabetes. These data make a compelling case for pharmacologic therapy as a foundation of long-term obesity care [2,3,4].

Yet real-world persistence is challenging. Large observational datasets show substantial discontinuation within the first year and relatively low rates of timely re-initiation; when therapy stops, weight regain is common, as documented in randomized withdrawal (STEP-4). Together, these findings underscore the adherence-sensitivity of clinical benefit and motivate strategies that make treatment more livable and sustainable [1,5].

Beyond the gut–pancreas axis, GLP-1 signaling acts in brain circuits that govern appetite, motivation, and reward. Human neuroimaging and behavioral studies, alongside contemporary systems-neuroscience work, indicate that GLP-1RAs dampen responsivity to palatable food cues and interact with mesolimbic dopamine pathways that sustain hedonic feeding. These central actions likely contribute to reduced cravings, smaller portion sizes, and easier caloric restriction for many patients [6].

At the same time, concerns have been raised about possible “hedonic blunting” or mood effects in a subset of users. Population-based analyses to date do not show an overall increase in suicidality with GLP-1RAs, and regulators (EMA/PRAC) have not established a causal link, but pharmacovigilance signals and case reports warrant ongoing surveillance and patient-centered discussion [7].

Intermittent fasting has emerged as a complementary, low-cost, and sustainable lifestyle intervention. Protocols such as alternate-day fasting, time-restricted feeding, and prolonged >24 h fasts activate distinct metabolic pathways, including enhanced fat oxidation, ketogenesis, autophagy, and insulin sensitization [1]. Importantly, unlike pharmacotherapy, intermittent fasting may preserve the intrinsic psychological dimension of food reward and life pleasure, while potentially offering protective effects against depression and anxiety in certain populations. However, it may also trigger irritability, binge–restrict cycles, or disordered eating in vulnerable groups [8].

Intermittent fasting/time-restricted eating and targeted lifestyle modification engage complementary biological levers—ketogenesis and fat oxidation, improved insulin sensitivity, and nutrient-sensing pathways such as Adenosine Monophosphate–Activated Protein Kinase (AMPK)—mammalian Target of Rapamycin (mTOR)—sirtuins —while also offering behavioral scaffolds (time boundaries, routine) that can reinforce pharmacotherapy. Syntheses of randomized trials and umbrella reviews suggest meaningful benefits for weight, glycemia, and cardiometabolic risk factors, with generally neutral or favorable effects on energy and mood; nonetheless, fasting paradigms can precipitate or exacerbate disordered-eating phenotypes in vulnerable individuals and must be applied with care [9].

The intersection of pharmacotherapy and lifestyle medicine is increasingly relevant. While GLP-1RA therapy reduces appetite and hedonic drive, intermittent fasting and structured lifestyle modification promote metabolic flexibility, muscle preservation through protein-focused diets and resistance training, and long-term patient empowerment. This synergy may improve outcomes and clinical durability. Moreover, cost-effectiveness considerations are paramount: GLP-1 RAs remain expensive and require ongoing administration, whereas intermittent fasting carries minimal direct costs but higher behavioral demands. Combining both strategies may optimize patient outcomes across metabolic, psychological, and economic domains [10].

The purpose of this review is to provide a comprehensive and integrative analysis of current evidence regarding the combined use of glucagon-like peptide-1 receptor agonist (GLP-1RA) therapy and intermittent fasting within the broader framework of lifestyle modification. By examining their complementary physiological, metabolic, and behavioral mechanisms, this review seeks to elucidate how the strategic alignment of pharmacological appetite regulation with structured nutritional and exercise interventions can enhance treatment outcomes, optimize body composition—particularly through preservation of lean mass—and extend cardiometabolic and longevity benefits. Furthermore, it aims to contextualize this hybrid approach within contemporary cost-effectiveness and patient-centered care paradigms, proposing a translational model for sustainable obesity and metabolic disease management.

## 2. Methods

This manuscript employs a narrative, integrative review aimed at synthesizing a pragmatic, long-term efficacy-oriented framework that integrates intermittent fasting and lifestyle modification with GLP-1RA therapy. We hypothesize that (i) aligning pharmacologic appetite suppression with structured eating windows can reduce decision fatigue, improve tolerability, and preserve efficacy during dose optimization; (ii) codifying protein-forward, resistance-training-inclusive plans can protect lean mass while supporting metabolic health; and (iii) embedding behavioral safeguards can mitigate psychological downsides and sustain long-term adherence to a healthier lifestyle. Our goal is to provide a framework for integrating these approaches into future clinical practice and research.

### 2.1. Literature Search Strategy

A structured—but not systematic—literature search was performed in PubMed, Web of Science, and the Cochrane Library (2010–July 2025). Searches used combinations of terms including: “GLP-1 receptor agonist,” “semaglutide,” “liraglutide,” “tirzepatide,” “intermittent fasting,” “time-restricted eating,” lifestyle modification,” “body composition,” “adherence,” “psychological impact,” “cost-effectiveness,” and “longevity.”

The search aimed to identify representative, clinically influential, and conceptually relevant studies rather than exhaustively catalog all existing research. Only English-language studies and human data were included. Bibliographies of relevant reviews were hand-searched to identify additional sources.

### 2.2. Selection Principles

Studies were selected for inclusion based on their relevance to key themes, methodological quality, and their ability to illuminate mechanistic, clinical, or behavioral aspects of GLP-1 therapy and fasting. Priority was given to:

- Randomized clinical trials
- Large observational cohorts
- Meta-analyses and systematic reviews
- Translational and mechanistic studies relevant to appetite, metabolism, or muscle biology
- Authoritative guidelines, advisories, and consensus statements

The evidence was synthesized iteratively based on relevance to the conceptual framework. Mechanistic studies were considered only when they were (1) published in peer-reviewed journals, (2) directly relevant to appetite regulation, metabolic adaptation, or muscle physiology, and (3) cited frequently or recognized as foundational within their respective research domains.

### 2.3. Synthesis Approach

The focus was on integrating findings, identifying converging evidence, and highlighting gaps requiring future study, rather than producing pooled quantitative estimates. Evidence was synthesized narratively, with thematic clustering into four domains:

- Efficacy & Adherence: GLP-1RA vs. intermittent fasting
- Psychological impact: mood, reward circuits, disordered eating
- Cost–Benefit analyses: direct and indirect health economics
- Longevity & mechanistic insights: nutrient sensing, metabolic adaptation.

## 3. Results/Evidence Synthesis

### 3.1. Efficacy and Adherence

GLP-1RAs such as semaglutide, liraglutide, and tirzepatide demonstrate substantial efficacy in promoting weight reduction, glycemic control, and cardiovascular benefit across diverse populations with obesity and type 2 diabetes. Clinical trials consistently show weight loss averaging 10–20% of baseline body weight with newer GLP-1 analogs, alongside significant improvements in HbA1c, lipid profiles, and reductions in cardiovascular events [2,3,11]. However, real-world adherence remains a recurring limitation, with discontinuation rates of up to 50% within two years, largely due to gastrointestinal side effects, diminished reward response to food, injection burden, and the high monthly cost [12].

Recent guidance from major professional societies underscores that GLP-1 therapy is most effective when paired with intentional nutritional planning. Appetite suppression and slower gastric emptying can reduce overall protein and micronutrient intake, making protein-forward, nutrient-dense meals essential for preserving lean mass and supporting metabolic health. At the same time, clinical recommendations highlight the importance of proactively managing common GLP-1–related gastrointestinal symptoms through gradual dose escalation, smaller meals, and limiting high-fat or highly processed foods. Together, these strategies improve tolerability, maintain adherence, and help sustain the metabolic benefits of GLP-1–based obesity treatment [13,14].

In contrast, intermittent fasting protocols—particularly time-restricted eating (e.g., 16:8) and alternate-day or prolonged fasting (>24 h)—achieve more modest weight reduction of approximately 3–8% over 6–12 months and are associated with improvements in insulin sensitivity, lipid profiles, and blood pressure [15,16]. Unlike intermittent prolonged fasting, which implies extreme or severe caloric deprivation with higher attrition, structured intermittent fasting offers moderate adherence rates owing to its cultural adaptability and behavioral flexibility. Nevertheless, sustainability remains variable, with attrition rates approaching 40–50% in some cohorts; while many participants report empowerment and increased autonomy, others struggle with hunger, social eating constraints, and long-term feasibility [17]

Large RCTs and DXA substudies (e.g., semaglutide STEP program) report greater reductions in fat mass than in lean body mass, but a measurable absolute loss of lean tissue accompanies most drug-induced weight loss [2]. Trials of tirzepatide (SURMOUNT and diabetes trials) show very large total weight losses with reductions in both fat and lean mass; some analyses note relative preservation of lean mass proportionally and reductions in intramuscular fat, which could favor muscle efficiency—but absolute lean-mass loss still occurs [3]. Systematic reviews and recent narrative reviews echo these findings and emphasize that most RCTs did not include robust functional endpoints (e.g., grip strength, 6 min walk) as primary outcomes, so the impact on physical function and frailty remains incompletely characterized [18,19].

**Figure 1:**
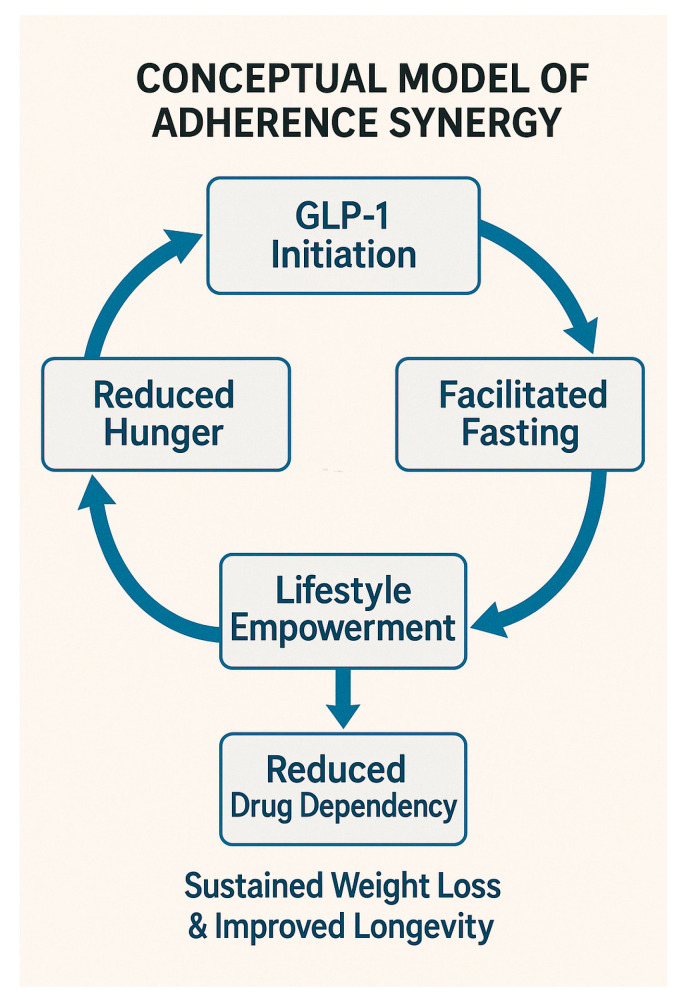
Conceptual model of improved outcomes through synergy of pharmacotherapy and lifestyle: The diagram summarizes hypothesized pathways—including reduced hunger, facilitated fasting, lifestyle empowerment, and decreased long-term drug dependency—leading to sustained weight loss and improved longevity.

Some studies of time-restricted eating, alternate-day fasting, and other intermittent fasting protocols consistently demonstrate modest weight loss with preferential reduction in fat mass, though lean mass decline still occurs, particularly in the absence of adequate protein intake or resistance training [20]. Some studies note preservation of lean body proportion relative to baseline weight, but absolute lean-mass reductions are measurable and may affect long-term functional health [21]. Evidence on functional outcomes is sparse: few studies assess muscle strength, endurance, or physical performance directly, and available data suggest that without exercise support, intermittent fasting alone does not reliably preserve functional capacity [22,23,24]. Figure 1 illustrates the conceptual model of synergy, highlighting how GLP-1 initiation reduces hunger and facilitates intermittent fasting, which in turn fosters lifestyle empowerment, decreases long-term drug dependency, and ultimately supports sustained weight loss and improved longevity.

Emerging evidence highlights that structured diet and physical activity remain essential complements to GLP-1 therapy. Appetite suppression can reduce overall protein and energy intake, which may contribute to lean-mass loss if not balanced with protein-rich meals and regular resistance exercise. Recent reviews also raise concern about potential sarcopenia during GLP-1–induced weight loss, underscoring the need to integrate nutrition planning and strength-focused activity to preserve muscle mass and functional capacity [25,26].

Recent evidence indicates that GLP-1 receptor agonists have complex effects on muscle health. While some emerging data suggest potential benefits for muscle quality and metabolic function, systematic reviews show that GLP-1 therapy is still associated with measurable reductions in lean mass, particularly in individuals with diabetes or low baseline muscle reserves [27]. These findings reinforce that sarcopenia risk remains clinically relevant during GLP-1–induced weight loss. In addition, contemporary clinical guidance emphasizes that managing gastrointestinal symptoms—through dose titration, smaller meals, and attention to dietary composition—is essential for maintaining tolerability and preventing excessive reductions in energy and protein intake, both of which are critical for preserving muscle mass [14,28].

When applied together, pharmacotherapy and lifestyle strategies may reinforce one another. GLP-1RAs blunt early hunger signals and reduce food cue reactivity, easing the entry into fasting windows, while structured fasting fosters self-efficacy, internal locus of control, and metabolic flexibility, potentially reducing long-term dependence on pharmacotherapy. Comparative data suggest that pharmacologic therapy produces more rapid and sustained weight loss, whereas intermittent fasting may enhance psychological acceptability and adherence [29]. Early pilot studies indicate that integration of these approaches may improve the durability of weight loss, lower required drug doses, and yield greater long-term adherence than either strategy alone [30].

GLP-1RA therapy may necessitate intentional dietary planning, as appetite suppression and reduced meal frequency can inadvertently lower protein intake and compromise lean mass preservation. When combined with intermittent fasting, structured feeding windows require prioritization of high-quality, protein-rich meals to stimulate muscle protein synthesis and maintain metabolic health. Incorporating resistance exercise alongside targeted nutrition further enhances anabolic signaling, mitigating the risk of sarcopenia while optimizing the therapeutic synergy of pharmacological and lifestyle interventions [30,31]. Although prioritizing high-quality protein intake is important for preserving lean mass during GLP-1RA therapy and intermittent fasting, achieving adequate protein consumption can be challenging in real-world practice. Appetite suppression from GLP-1RAs, combined with restricted feeding windows during fasting protocols, may limit meal size and reduce opportunities to consume sufficient protein. These constraints highlight the need for intentional meal planning, structured guidance on protein distribution within eating windows, and, when appropriate, support from nutrition professionals. Rather than implying effortless adherence, this recommendation reflects an aspirational clinical target aimed at mitigating lean-mass loss and supporting functional health [25,26].

### 3.2. Psychological Impact

The psychological effects of GLP-1RAs and intermittent fasting are increasingly recognized as key determinants of adherence and long-term success. GLP-1 therapy reduces hedonic eating by attenuating hypothalamic and mesolimbic dopaminergic signaling, thereby suppressing reward-driven food intake [32].

While this mechanism facilitates weight loss and reduces compulsive overeating, it may also blunt the pleasure associated with eating, diminish social enjoyment of meals, and, in some individuals, contribute to dysphoria or depressive symptoms [33]. These observations underscore the importance of psychological support for patients vulnerable to mood disturbances during long-term pharmacotherapy.

Intermittent fasting protocols exert distinct yet complementary psychological influences. Early fasting phases may be characterized by irritability, hunger, and preoccupation with food, but adaptive metabolic switching and ketosis can subsequently enhance mood stability, cognitive clarity, and resilience [34]. Ketone bodies have been shown to augment central GABAergic tone, potentially buffering stress reactivity, while refeeding phases restore hedonic satisfaction and preserve the cultural and emotional role of food [35,36]. Structured fasting cycles may therefore maintain life satisfaction and sense of control more effectively than continuous pharmacologic suppression of appetite. Importantly, when fasting is paired with nutrient-dense, protein-rich dietary strategies, risks of mood decline, fatigue, or disordered eating are minimized [37].

Head-to-head conceptual comparisons suggest that GLP-1 therapy is more effective at suppressing compulsive overeating and dampening food cue reactivity, while intermittent fasting may better sustain psychological well-being, self-efficacy, and internal locus of control [38]. The convergence of these approaches may offer a synergistic solution: GLP-1RAs reduce physiological hunger and ease adherence to fasting windows, while structured fasting preserves hedonic eating within defined cycles and mitigates the emotional flatness sometimes associated with chronic pharmacotherapy. Future research should clarify whether this bidirectional support system minimizes psychological risks while optimizing adherence and long-term quality of life.

### 3.3. Cost–Benefit Analyses

GLP-1 receptor agonists represent one of the most effective but also one of the most expensive pharmacotherapies for obesity and type 2 diabetes. Annual costs typically range from $4000 to $12,000 per patient in high-income settings, depending on formulation and insurance coverage, with many healthcare systems reporting yearly expenditures exceeding $8000 [39]

Cost-effectiveness analyses demonstrate that GLP-1 therapy can be justified when accounting for downstream reductions in diabetes complications, cardiovascular events, and improvements in quality-adjusted life years (QALYs). However, the need for continuous administration and long-term affordability remains a major barrier at the population scale, limiting accessibility in routine practice [10].

In contrast, intermittent fasting carries negligible direct cost, though successful implementation requires behavioral support, education, and monitoring to minimize risks of disordered eating. Indirect benefits include reduced healthcare utilization, lower medication burden, improved productivity, and enhanced patient empowerment, making lifestyle-based approaches broadly accessible and scalable [9,40]. Table 1 provides a conceptual comparison of the economic and scalability considerations associated with GLP-1RA monotherapy, intermittent fasting, and a potential combined hybrid approach.

At present, no studies have formally evaluated the cost-effectiveness of combining GLP-1RA therapy with intermittent fasting. Any discussion of potential synergy is therefore conceptual and must be interpreted cautiously. Theoretically, short-term pharmacologic therapy may accelerate early weight loss and metabolic improvements, while structured fasting could lower long-term medication dependence and overall treatment cost. Yet these hypotheses remain untested. Future modeling studies and prospective trials are needed to determine whether integrative protocols can achieve durable health benefits while reducing lifetime pharmacotherapy exposure and total healthcare expenditures.

### 3.4. Longevity and Mechanistic Insights

Beyond short-term efficacy, both GLP-1RAs and intermittent fasting have implications for longevity and healthy aging through modulation of complementary biological pathways. GLP-1 therapy confers cardiometabolic protection, reduces systemic inflammation, and improves endothelial function, thereby lowering mortality risk [32]. Preclinical studies further suggest neuroprotective effects, with GLP-1 analogues enhancing synaptic plasticity, improving insulin signaling in the brain, and attenuating Alzheimer’s-related pathology by reducing amyloid deposition [43].

Intermittent fasting protocols, by contrast, activate nutrient-sensing pathways such as AMPK, sirtuins, and mTOR, thereby promoting autophagy, mitochondrial biogenesis, DNA repair, and oxidative stress resistance [37,44,45]. Human translational data link fasting to reduced circulating IGF-1, enhanced stem cell regeneration, and improved cardiometabolic resilience [46].

Unlike prolonged fasting windows, which risk nutrient deficiency and catabolism, structured fasting appears to promote metabolic flexibility and resilience, especially when combined with resistance exercise and protein-rich dietary strategies that mitigate lean mass loss often associated with both caloric restriction and pharmacotherapy [47].

The conceptual synthesis suggests that integrating GLP-1 therapy with structured intermittent fasting may provide synergistic benefits for healthspan and lifespan. GLP-1RAs primarily target vascular, metabolic, and neuroprotective pathways, while fasting augments cellular stress resistance, mitochondrial renewal, and systemic rejuvenation. Together, these interventions could extend not only years of life but also years of healthy life, representing a promising avenue for translational longevity strategies [48]. Table 2 provides a comparative overview of the mechanistic, clinical, psychological, and safety dimensions of GLP-1 receptor agonists, intermittent fasting, and a proposed combined approach.

Table 3 consolidates key clinical investigations referenced in the review, outlining study populations, intervention characteristics, duration, and primary metabolic or body-composition outcomes. It also provides a comparative foundation for interpreting the physiological and clinical claims developed throughout the manuscript.

## 4. Discussion and Future Directions

The present synthesis highlights the potential added value of integrating intermittent fasting with GLP-1RA therapy in the management of obesity and type 2 diabetes. GLP-1RAs remain among the most effective pharmacological interventions, delivering robust weight reduction, glycemic control, and cardiovascular protection [2,3,4]. RCT evidence supports that GLP-1RAs induce lean-mass reductions proportional to weight loss and highlights an urgent need for trials that simultaneously measure body composition and functional outcomes and test mitigation strategies (protein-targeted nutrition + resistance exercise) as well as the need for integrative protocols that combine fasting with protein-optimized diets and resistance exercise to safeguard muscle function.

However, GLP-1RA long-term effectiveness is frequently constrained by gastrointestinal side effects, high cost, and declining adherence over time. Conversely, intermittent fasting and structured lifestyle modifications demonstrate physiological and psychological benefits, including improved insulin sensitivity, lipid regulation, mood stabilization, and enhanced self-efficacy, yet they are often limited by inconsistent adherence and social or practical challenges [12,15].

Optimizing dietary composition is critical when combining GLP-1RA therapy with intermittent fasting to preserve lean mass and maximize metabolic outcomes. GLP-1RAs effectively reduce appetite and slow gastric emptying, which can inadvertently decrease overall protein intake, placing patients at risk for muscle loss if nutritional adequacy is not maintained. Intermittent fasting introduces additional catabolic stress during fasting windows, though metabolic adaptations such as ketosis and AMPK activation can help preserve energy efficiency [61]. To mitigate lean mass loss, protein-rich meals should be prioritized during feeding windows, ideally early in the cycle, with emphasis on leucine-rich sources to enhance muscle protein synthesis. Concurrent resistance exercise further supports anabolic signaling, while adequate carbohydrate and healthy fat intake timed around physical activity and refeeding phases ensures energy availability and adherence [60]. Strategic alignment of macronutrient intake, feeding timing, and exercise thus provides a synergistic framework, enabling patients to achieve the metabolic and weight-loss benefits of both GLP-1RA therapy and intermittent fasting while minimizing adverse effects on muscle massIntegrating these approaches leverages complementary mechanisms: GLP-1Ras reduce early hunger signals, facilitating adherence to fasting windows, while intermittent fasting provides behavioral scaffolding, enhances internal locus of control, and may mitigate the blunting of hedonic reward observed with chronic GLP-1 therapy. Economically, hybrid approaches may optimize cost-effectiveness by reducing long-term drug exposure while maintaining metabolic benefits. Mechanistically, GLP-1Ras target vascular, metabolic, and neuroprotective pathways, whereas fasting activates cellular stress responses, autophagy, mitochondrial biogenesis, and nutrient-sensing pathways (AMPK, sirtuins, mTOR), offering convergent pathways toward improved health span and longevity [38,41,46].

Although GLP-1 receptor agonists and intermittent fasting may offer complementary metabolic benefits, the potential for overlapping adverse effects must be acknowledged. GLP-1RAs commonly produce gastrointestinal symptoms such as nausea, early satiety, and reflux, and these effects could theoretically be intensified when combined with prolonged fasting windows, especially during dose escalation or in individuals with low caloric intake. Because no clinical trials have directly evaluated the safety of this combined approach, any discussion of synergy remains conceptual. As such, individualized fasting schedules, gradual medication titration, and careful monitoring are essential to minimize discomfort and ensure tolerability. Future studies will be required to determine whether combined protocols enhance benefits without exacerbating side effects.

### 4.1. Clinical Translation and Research Directions

Personalized protocols are essential, as patient tolerance, access to pharmacotherapy, and lifestyle feasibility vary. GLP-1RA initiation may serve as a bridge to intermittent fasting and structured lifestyle interventions, with fasting maintaining long-term benefits after pharmacotherapy tapering.

Future research should prioritize:

- Prospective, controlled trials comparing GLP-1 monotherapy, fasting/lifestyle interventions alone, and combined regimens. Future RCTs should track lean mass and functional outcomes, not just weight and glycemia, especially when combining GLP-1Ras with fasting.
- Neurobehavioral studies to elucidate interactions between hedonic regulation under GLP-1 therapy and the restorative pleasure of structured refeeding during fasting cycles. Long-term real-world effectiveness studies, particularly examining adherence, quality of life, and economic outcomes.
- Integrative mechanistic studies assessing how pharmacological and lifestyle interventions synergize at cellular, metabolic, and neurobehavioral levels to enhance longevity and cardiometabolic resilience.
- Longevity studies evaluating fasting-anchored maintenance protocols for long-term cardiometabolic risk reduction and functional independence in aging populations.

### 4.2. Proposed Stepwise Hybrid Model

**Figure 2:**
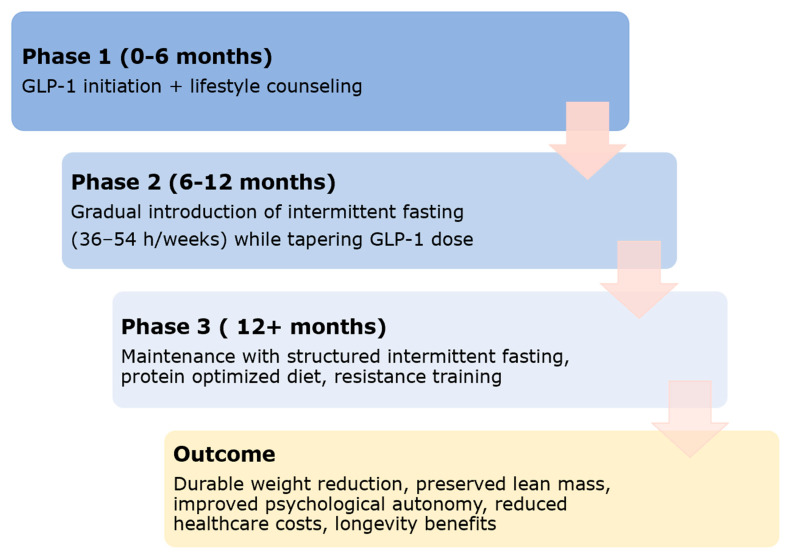
Phased Integration of GLP-1 Therapy and Intermittent Fasting for Sustainable Weight and Metabolic Health: This figure presents a schematic, conceptual model outlining a proposed phased approach for integrating GLP-1 receptor agonist therapy with structured intermittent fasting and lifestyle interventions. The diagram is intended to illustrate theoretical clinical sequencing and mechanistic rationale.

The staged framework we propose represents a novel translational model for integrating GLP-1RA therapy with intermittent fasting. By sequencing pharmacological initiation, structured fasting transition, and lifestyle-based maintenance, this approach addresses the well-documented limitations of long-term GLP-1RA adherence while leveraging their appetite-suppressing effects to ease the adoption of fasting protocols. Unlike prior studies that examined pharmacotherapy or lifestyle modification in isolation, this model highlights a synergistic pathway that may optimize weight durability, preserve lean mass when combined with protein intake and resistance exercise, and activate complementary longevity mechanisms. Future randomized controlled trials are warranted to test this phased strategy against monotherapy approaches, with outcomes extending beyond weight loss to include metabolic resilience, functional capacity, quality of life, and cost-effectiveness. This framework (Figure 2) could yield durable clinical outcomes, reduce healthcare expenditures, and preserve patient autonomy by limiting lifelong dependence on pharmacotherapy. By uniting the pharmacological potency of GLP-1 therapy with the evolutionary resilience of intermittent fasting, clinicians may unlock a novel paradigm in obesity and metabolic disease care—one that is effective, affordable, and longevity-oriented.

### 4.3. Limitations of Current Evidence

The literature is limited by heterogeneity in intermittent fasting protocols, short follow-up periods, and a paucity of data on combined GLP-1RA–fasting strategies. Cost-effectiveness analyses largely rely on modeling assumptions, while psychological outcomes remain underreported in pharmacotherapy trials. Addressing these gaps will require comprehensive frameworks that integrate physiological, behavioral, and economic endpoints.

A key limitation of this review is the absence of direct clinical trials evaluating the combined use of GLP-1 receptor agonists and intermittent fasting. Our proposed hybrid framework is therefore based on extrapolations from separate bodies of evidence examining each intervention independently, including their metabolic, behavioral, and physiological effects. While this conceptual synthesis is grounded in established mechanisms and consistent findings across related studies, it should not be interpreted as evidence of proven synergy. Future randomized and longitudinal trials are needed to formally assess the safety, efficacy, and long-term durability of integrated GLP-1RA–fasting approaches.

## 5. Conclusions

GLP-1RA and intermittent fasting represent distinct yet complementary strategies for managing obesity. Pharmacotherapy provides rapid, potent, and clinically validated efficacy, whereas lifestyle modification offers low-cost, sustainable, and psychologically rewarding pathways for long-term health. When integrated, these strategies may enhance outcomes, mitigate adverse psychological effects, reduce overall healthcare costs, and engage convergent mechanisms promoting longevity and metabolic resilience.

This integrated strategy is a promising conceptual model not only to optimize individual patient outcomes but also to transform public health strategies for obesity and metabolic disease; however, evidence for their combined use remains indirect. Current data support the biological plausibility and practical rationale for aligning pharmacologic and lifestyle strategies; nevertheless, definitive conclusions about their synergistic efficacy cannot yet be drawn. Well-designed clinical trials are needed to evaluate combined protocols, determine long-term safety and adherence, and clarify their potential to enhance metabolic health and durability of weight loss.

## Figures and Tables

**Table 1:** Cost–Benefit Framework.

Factor	GLP-1 Monotherapy	Intermittent Fasting	Combined Hybrid Approach
Direct cost	$800–$1200/month (US prices, variable by country) [41]	Minimal (nutritional counseling, monitoring) [9,40]	Initial high cost, then reduced drug dose → cost savings
Indirect cost	Possible lifelong therapy [10,41]	Minimal [9,40]	Reduced long-term burden of chronic disease
Healthcare savings	Significant if adherence maintained, but limited by discontinuation	Substantial if adherence high	Maximized: early drug-driven improvements consolidated by fasting
Scalability	Limited by healthcare budgets & insurance [10]	Highly scalable, low infrastructure needed [9,40]	Balanced, scalable after initial drug-supported adaptation
Cost-effectiveness (per QALY)	Cost-effective in high-risk populations but questionable for primary prevention [42]	Very cost-effective due to negligible cost [9,40]	Most promising: optimized clinical outcomes with reduced costs

**Table 2:** Comparative Framework: GLP-1 Therapy vs. Intermittent Fasting vs. Combined Strategy.

Dimension	GLP-1 Receptor Agonists	Intermittent Fasting (36–54 h/Week)	Combined Approach
Primary mechanism	GLP-1 receptor activation → appetite suppression, delayed gastric emptying, improved insulin secretion [49]	Nutrient deprivation → ketogenesis, autophagy, improved insulin sensitivity, circadian alignment [48]	Synergistic appetite control + metabolic remodeling
Weight loss efficacy	High (10–20% in RCTs) but plateaus with time [2,3]	Moderate (5–12%), more gradual, depends on adherence [50]	Potentially additive; faster onset with GLP-1, durable maintenance with fasting
Lean mass preservation	Variable, risk of muscle loss without resistance training/protein intake [51]	Better preservation when fasting combined with protein and exercise [52]	Optimized when protein & exercise are integrated into hybrid program
Psychological impact	Reduced food reward, possible blunting of pleasure & mood (depression risk) [53]	Enhanced self-control, improved stress resilience; hunger manageable after adaptation [54]	May balance pharmacological suppression with empowerment of voluntary control
Adherence profile	Often declines after 12–18 months; cost barrier [55]	Requires initial adaptation, improves with structured programs [56]	GLP-1 facilitates entry, fasting provides long-term sustainability
Safety profile	GI side effects, gallbladder risk, cost constraints [57]	Risk of hypoglycemia (in diabetics on insulin), transient headaches/fatigue [36]	Lower drug dose needed, fewer pharmacological side effects
Longevity impact	Unclear; modest cardiometabolic benefits [43,58]	Strong mechanistic evidence (autophagy, inflammation reduction, mitochondrial renewal) [59]	May extend healthspan by merging short-term drug efficacy with long-term fasting biology

**Table 3:** Summary of Principal Clinical Studies Informing the Mechanistic and Therapeutic Framework.

Study	Study Type	Population (N)	Study Duration	Intervention/Therapy	Fasting Protocol (If Applicable)	Key Outcomes
Wilding et al., 2021 (STEP-1) [2]	Randomized, double-blind, placebo-controlled multicenter trial	1961 adults with obesity or overweight (BMI ≥ 27 + ≥1 comorbidity), without diabetes	68 weeks	Semaglutide 2.4 mg weekly + lifestyle intervention (−500 kcal/day diet, ≥150 min/week physical activity) vs. placebo	None	−14.9% mean weight loss; improved cardiometabolic markers; high rates of reversion from prediabetes to normoglycemia
Jastreboff et al., 2022 (SURMOUNT-1) [3]	Randomized, double-blind, placebo-controlled, phase 3 trial	2539 adults with obesity or overweight (BMI ≥ 27 with ≥1 comorbidity), without diabetes	72 weeks	Tirzepatide 5–15 mg weekly + lifestyle intervention vs. placebo + lifestyle	None	Up to −20.9% weight loss; up to 57% achieving ≥20% loss; improvements in lipids, glycemia, waist circumference, prediabetes reversion, and quality-of-life scores
Lincoff et al., 2023 (SELECT) [4]	Randomized, double-blind, placebo-controlled cardiovascular outcomes trial	17,604 adults with overweight/obesity (BMI ≥ 27) & CVD, no diabetes	Median 40 months	Semaglutide 2.4 mg weekly	None	−20% relative risk reduction in MACE; weight loss (~9.4%); reductions in HbA1c, CRP, inflammatory markers;
Rubino et al., 2021 (STEP-4) [5]	Randomized, double-blind, withdrawal, placebo-controlled trial	803 adults with overweight (BMI ≥27 + comorbidity)/obesity; no diabetes	20 weeks run-in + 48 weeks randomized phase	20-week semaglutide run-in, then randomized to continue semaglutide vs. switch to placebo (both with lifestyle intervention)	None	Continuation preserved weight loss; discontinuation led to rapid weight regain;
Rodriguez et al., 2025 [1]	Real-world, retrospective cohort using U.S. electronic health records	>30,000 adults with overweight/obesity	Not fixed	GLP-1RA use patterns: discontinuation & reinitiation	None	High GLP-1RA discontinuation rates; very low reinitiation; significant adherence challenges in routine clinical practice;
Stec et al., 2023 (Nutrients) [8]	Prospective interventional study	40 middle-aged men	8 days fasting	8-day medically supervised fast	8-day prolonged fasting protocol	Weight loss, ↓ BP, ↑ mood; no major adverse events reported
Gabel et al., 2018 [56]	Randomized controlled trial	23 adults with obesity	12 weeks	Time-restricted eating (TRE) with an 8 h eating window	TRE 8:16 (eat 10:00–18:00, fast 16 h)	−2.6% weight loss; improved BP; variable adherence
Keenan et al., 2022 [60]	Randomized controlled trial	41 exercise-trained adults	12 weeks	TRE vs. continuous restriction + resistance training	TRE 16:8 (16 h fast, 8 h eating window)	Similar fat loss; preservation of muscle strength and lean mass with resistance training; TRE did not impair performance or adaptation.
Xie et al., 2024 [22]	Systematic review of randomized controlled trials	13 RCTs	-	Time-restricted eating interventions	TRE 8–12 h/day	Fat loss dependent on window duration; modest lean-mass loss in some trials
Kazeminasab et al., 2025 [24]	Meta-analysis of randomized and non-randomized studies	23 studies	-	Intermittent fasting & calorie restriction	ADF, TRE, modified fasting protocols	No consistent improvements in strength performance; variable adherence
Johnson et al., 2025 [31]	Cross-sectional study	263 adults on GLP-1RAs	-	Assessment of nutrient intake during GLP-1RA therapy	None	Lower protein and micronutrient intake was common; risk of lean-mass loss
Sandsdal et al., 2023 [30]	Randomized controlled trial	92 adults with metabolic syndrome	16 weeks	Exercise + GLP-1RA vs. GLP-1RA alone	None	Combination therapy led to greater reductions in abdominal fat and improved metabolic syndrome severity compared with GLP-1RA alone
Hwang et al., 2025 [10]	Lifetime health-economic simulation model	US adults with obesity	Lifetime simulation	Semaglutide vs. tirzepatide (cost-effectiveness and long-term health outcomes)	None	Both cost-effective in high-risk groups; long-term affordability uncertain
Pantanetti et al., 2024 [51]	Real-world observational study	164 adults with T2D	6 months	Semaglutide therapy in routine clinical practice	None	Significant weight & fat mass loss; measurable lean-mass reduction
Moro et al., 2016 [52]	Randomized controlled trial	34 resistance-trained males	8 weeks	Time-restricted feeding combined with resistance training	TRE 16:8 (16 h fast, 8 h eating window)	↓ fat mass, maintained muscle strength, ↓ inflammation
Stekovic et al., 2019 [17]	Randomized controlled trial	60 healthy adults	4 weeks	Alternate-day fasting intervention	ADF (36 h fast alternated with feeding days)	Improved BP, lipid profile; ↑ ketones; modest weight loss;good overall tolerability

ADF = alternate-day fasting, BMI = body mass index, BP = blood pressure, CRP = C reactive protein, CVD = cardiovascular disease, HbA1C = glycated hemoglobin, MACE = major adverse cardiovascular events, TRE = time restricted eating, T2D = type 2 diabetes.

## Data Availability

Not applicable.

## Abbreviations

The following abbreviations are used in this manuscript:

Abbreviation	Full Term/Definition
AMPK	Adenosine Monophosphate–Activated Protein Kinase
BMI	Body Mass Index
CGM	Continuous Glucose Monitoring
EMA/PRAC	European Medicines Agency/Pharmacovigilance Risk Assessment Committee
GI	Gastrointestinal
GLP-1	Glucagon-Like Peptide-1
GLP-1RA	Glucagon-Like Peptide-1 Receptor Agonist
HbA1c	Glycated Hemoglobin
IGF-1	Insulin-Like Growth Factor-1
IF	Intermittent Fasting
mTOR	Mammalian Target of Rapamycin
QALY	Quality-Adjusted Life Year
RA	Receptor Agonist
RCT	Randomized Controlled Trial
TRE	Time-Restricted Eating
T2DM	Type 2 Diabetes Mellitus

## References

[1] Rodriguez P.J., Zhang V., Gratzl S., Do D., Goodwin Cartwright B., Baker C., Gluckman T.J., Stucky N., Emanuel E.J. (2025). Discontinuation and Reinitiation of Dual-Labeled GLP-1 Receptor Agonists Among US Adults with Overweight or Obesity. JAMA Netw. Open.

[2] Wilding J.P.H., Batterham R.L., Calanna S., Davies M., Van Gaal L.F., Lingvay I., McGowan B.M., Rosenstock J., Tran M.T.D., Wadden T.A. (2021). Once-Weekly Semaglutide in Adults with Overweight or Obesity. N. Engl. J. Med..

[3] Jastreboff A.M., Aronne L.J., Ahmad N.N., Wharton S., Connery L., Alves B., Kiyosue A., Zhang S., Liu B., Bunck M.C. (2022). Tirzepatide Once Weekly for the Treatment of Obesity. N. Engl. J. Med..

[4] Lincoff A.M., Brown-Frandsen K., Colhoun H.M., Deanfield J., Emerson S.S., Esbjerg S., Hardt-Lindberg S., Hovingh G.K., Kahn S.E., Kushner R.F. (2023). Semaglutide and Cardiovascular Outcomes in Obesity without Diabetes. N. Engl. J. Med..

[5] Rubino D., Abrahamsson N., Davies M., Hesse D., Greenway F.L., Jensen C., Lingvay I., Mosenzon O., Rosenstock J., Rubio M.A. (2021). Effect of Continued Weekly Subcutaneous Semaglutide vs Placebo on Weight Loss Maintenance in Adults with Overweight or Obesity: The STEP 4 Randomized Clinical Trial. JAMA.

[6] Jones L.A., Brierley D.I. (2025). GLP-1 and the Neurobiology of Eating Control: Recent Advances. Endocrinology.

[7] Chen J., Zhang Q., Wu Q., Zhang X., Xiang Z., Zhu S., Dai T., Si Y. (2025). Impact of GLP-1 Receptor Agonists on Suicide Behavior: A Meta-Analysis Based on Randomized Controlled Trials. J. Diabetes.

[8] Stec K., Pilis K., Pilis W., Dolibog P., Letkiewicz S., Głębocka A. (2023). Effects of Fasting on the Physiological and Psychological Responses in Middle-Aged Men. Nutrients.

[9] Song D.K., Kim Y.W. (2023). Beneficial effects of intermittent fasting: A narrative review. J. Yeungnam Med. Sci..

[10] Hwang J.H., Laiteerapong N., Huang E.S., Kim D.D. (2025). Lifetime Health Effects and Cost-Effectiveness of Tirzepatide and Semaglutide in US Adults. JAMA Health Forum.

[11] Marso S.P., Daniels G.H., Brown-Frandsen K., Kristensen P., Mann J.F.E., Nauck M.A., Nissen S.E., Pocock S., Poulter N.R., Ravn L.S. (2016). Liraglutide and Cardiovascular Outcomes in Type 2 Diabetes. N. Engl. J. Med..

[12] Weiss T., Carr R.D., Pal S., Yang L., Sawhney B., Boggs R., Rajpathak S., Iglay K. (2020). Real-World Adherence and Discontinuation of Glucagon-Like Peptide-1 Receptor Agonists Therapy in Type 2 Diabetes Mellitus Patients in the United States. Patient Prefer. Adherence.

[13] Mozaffarian D., Agarwal M., Aggarwal M., Alexander L., Apovian C.M., Bindlish S., Bonnet J., Butsch W.S., Christensen S., Gianos E. (2025). Nutritional priorities to support GLP-1 therapy for obesity: A joint Advisory from the American College of Lifestyle Medicine, the American Society for Nutrition, the Obesity Medicine Association, and The Obesity Society. Am. J. Clin. Nutr..

[14] Kushner R.F., Almandoz J.P., Rubino D.M. (2025). Managing Adverse Effects of Incretin-Based Medications for Obesity. JAMA.

[15] Patterson R.E., Sears D.D. (2017). Metabolic Effects of Intermittent Fasting. Annu. Rev. Nutr..

[16] Varady K.A., Cienfuegos S., Ezpeleta M., Gabel K. (2022). Clinical application of intermittent fasting for weight loss: Progress and future directions. Nat. Rev. Endocrinol..

[17] Stekovic S., Hofer S.J., Tripolt N., Aon M.A., Royer P., Pein L., Stadler J.T., Pendl T., Prietl B., Url J. (2019). Alternate Day Fasting Improves Physiological and Molecular Markers of Aging in Healthy, Non-obese Humans. Cell Metab..

[18] Jobanputra R., Sargeant J.A., Almaqhawi A., Ahmad E., Arsenyadis F., Webb D.R., Herring L.Y., Khunti K., Davies M.J., Yates T. (2023). The effects of weight-lowering pharmacotherapies on physical activity, function and fitness: A systematic review and meta-analysis of randomized controlled trials. Obes. Rev..

[19] Jakicic J.M., Rogers R.J. (2025). The Role of Exercise in the Contemporary Era of Obesity Management Medications. Curr. Sports Med. Rep..

[20] Khalafi M., Maleki A.H., Ehsanifar M., Symonds M.E., Rosenkranz S.K. (2025). Longer-term effects of intermittent fasting on body composition and cardiometabolic health in adults with overweight and obesity: A systematic review and meta-analysis. Obes. Rev..

[21] Lowe D.A., Wu N., Rohdin-Bibby L., Moore A.H., Kelly N.T., Liu Y., Plodkowski R.A., Olapeju B., Keating K.D., Aspry K. (2020). Effects of Time-Restricted Eating on Weight Loss and Other Metabolic Parameters in Women and Men with Overweight and Obesity: The TREAT Randomized Clinical Trial. JAMA Intern. Med..

[22] Xie Y., Zhou K., Shang Z., Bao D., Zhou J. (2024). The Effects of Time-Restricted Eating on Fat Loss in Adults with Overweight and Obese Depend upon the Eating Window and Intervention Strategies: A Systematic Review and Meta-Analysis. Nutrients.

[23] Williamson E., Moore D.R. (2021). A Muscle-Centric Perspective on Intermittent Fasting: A Suboptimal Dietary Strategy for Supporting Muscle Protein Remodeling and Muscle Mass?. Front. Nutr..

[24] Kazeminasab F., Sharafifard F., Bahrami Kerchi A., Bagheri R., Carteri R.B., Kirwan R., Santos H.O., Dutheil F. (2025). Effects of Intermittent Fasting and Calorie Restriction on Exercise Performance: A Systematic Review and Meta-Analysis. Nutrients.

[25] Mehrtash F., Dushay J., Manson J.A.E. (2025). Integrating Diet and Physical Activity When Prescribing GLP-1s-Lifestyle Factors Remain Crucial. JAMA Intern. Med..

[26] Pantazopoulos D., Gouveri E., Papazoglou D., Papanas N. (2025). GLP-1 receptor agonists and sarcopenia: Weight loss at a cost? A brief narrative review. Diabetes Res. Clin. Pract..

[27] González-Luis A., Llinares-Arvelo V., Martínez-Alberto C.E., Hernández-Carballo C., Mora-Fernández C., Navarro-González J.F., Donate-Correa J. (2025). Glucagon-like peptide-1 receptor agonists and muscle health: Potential role in sarcopenia prevention and treatment. Eur. J. Endocrinol..

[28] Chen W., Qin H., Zhou Z., Lu Q., Li J., Zhang Y., Xu T., Wang L., Tang Y., Liu S. (2025). Glucagon-like peptide-1 receptor agonists and sarcopenia-related markers in diabetes: A systematic review and meta-analysis. Clin. Nutr..

[29] Chu J., Zhang H., Wu Y., Huang Y., Zhu T., Zhou Z., Wang H. (2025). Efficacy of lifestyle modification combined with GLP-1 receptor agonists on body weight and cardiometabolic biomarkers in individuals with overweight or obesity: A systematic review and meta-analysis. eClinicalMedicine.

[30] Sandsdal R.M., Juhl C.R., Jensen S.B.K., Lundgren J.R., Janus C., Blond M.B., Rosenkilde M., Bogh A.F., Gliemann L., Jensen J.-E.B. (2023). Combination of exercise and GLP-1 receptor agonist treatment reduces severity of metabolic syndrome, abdominal obesity, and inflammation: A randomized controlled trial. Randomized Control. Trial.

[31] Johnson B., Milstead M., Thomas O., McGlasson T., Green L., Kreider R., Jones R. (2025). Investigating nutrient intake during use of glucagon-like peptide-1 receptor agonist: A cross-sectional study. Front. Nutr..

[32] Nauck M.A., Quast D.R., Wefers J., Meier J.J. (2021). GLP-1 receptor agonists in the treatment of type 2 diabetes—State-of-the-art. Mol. Metab..

[33] Knudsen L.B., Lau J. (2019). The Discovery and Development of Liraglutide and Semaglutide. Front. Endocrinol..

[34] Longo V.D., Mattson M.P. (2014). Fasting: Molecular mechanisms and clinical applications. Cell Metab..

[35] Mattson M.P., Longo V.D., Harvie M. (2017). Impact of intermittent fasting on health and disease processes. Ageing Res. Rev..

[36] Anton S.D., Moehl K., Donahoo W.T., Marosi K., Lee S.A., Mainous A.G., Leeuwenburgh C., Mattson M.P. (2018). Flipping the Metabolic Switch: Understanding and Applying the Health Benefits of Fasting. Obesity.

[37] Brandhorst S., Longo V.D. (2019). Dietary Restrictions and Nutrition in the Prevention and Treatment of Cardiovascular Disease. Circ. Res..

[38] Koide Y., Kato T., Hayashi M., Daido H., Maruyama T., Ishihara T., Nishimura K., Tsunekawa S., Yabe D. (2025). Association between eating behavior patterns and the therapeutic efficacy of GLP-1 receptor agonists in individuals with type 2 diabetes: A multicenter prospective observational study. Front. Clin. Diabetes Healthc..

[39] Parker E.D., Lin J., Mahoney T., Ume N., Yang G., Gabbay R.A., ElSayed N.A., Bannuru R.R. (2024). Economic Costs of Diabetes in the U.S. in 2022. Diabetes Care.

[40] van den Burg E.L., van Peet P.G., Schoonakker M.P., Esmeijer A.C., Lamb H.J., Numans M.E., Pijl H., van den Akker-van Marle M.E. (2025). Cost-effectiveness of a periodic fasting-mimicking diet programme in patients with type 2 diabetes: A trial-based analysis and a lifetime model-based analysis. BMC Prim. Care.

[41] Hu Y., Zheng S.-L., Ye X.-L., Shi J.-N., Zheng X.-W., Pan H.-S., Zhang Y.-W., Yang X.-L., Huang P. (2022). Cost-effectiveness analysis of 4 GLP-1RAs in the treatment of obesity in a US setting. Ann. Transl. Med..

[42] Kim N., Vekeman F., Haluzík M., Dulal S., Tuttle R., Pang J., Wang F., Cohen J., McAdam-Marx C., Trueman P. (2022). Cost-effectiveness analysis of semaglutide 2.4 mg for the treatment of adult patients with overweight and obesity in the United States. J. Manag. Care Spec. Pharm..

[43] Hölscher C. (2014). Central effects of GLP-1: New opportunities for treatments of neurodegenerative diseases. J. Endocrinol..

[44] Brandhorst S., Longo V.D. (2016). Fasting and Caloric Restriction in Cancer Prevention and Treatment. Recent Results Cancer Res..

[45] Fontana L., Partridge L., Longo V.D. (2010). Extending healthy life span--from yeast to humans. Science.

[46] Choi I.Y., Lee C., Longo V.D., Mattson M.P., de Cabo R. (2016). A Diet Mimicking Fasting Promotes Regeneration and Reduces Autoimmunity and Multiple Sclerosis Symptoms. Cell Rep..

[47] Batsis J.A., Villareal D.T. (2018). Sarcopenic obesity in older adults: Aetiology, epidemiology and treatment strategies. Nat. Rev. Endocrinol..

[48] de Cabo R., Mattson M.P. (2019). Effects of Intermittent Fasting on Health, Aging, and Disease. N. Engl. J. Med..

[49] TMüller D., Finan B., Bloom S.R., D’Alessio D., Drucker D.J., Flatt P.R., Fritsche A., Gribble F., Grill H.J., Habener J.F. (2019). Glucagon-like peptide 1 (GLP-1). Mol. Metab..

[50] Harris L., Hamilton S., Azevedo L.B., Olajide J., De Brún C., Waller G., Whittaker V., Sharp T., Adams E., Ransom A. (2018). Intermittent fasting interventions for treatment of overweight and obesity in adults: A systematic review and meta-analysis. JBI Database Syst. Rev. Implement. Rep..

[51] Pantanetti P., Cangelosi G., Alberti S., Di Marco S., Michetti G., Cerasoli G., Di Giacinti M., Coacci S., Francucci N., Petrelli F. (2024). Changes in body weight and composition, metabolic parameters, and quality of life in patients with type 2 diabetes treated with subcutaneous semaglutide in real-world clinical practice. Front. Endocrinol..

[52] Moro T., Tinsley G., Bianco A., Marcolin G., Pacelli Q.F., Battaglia G., Palma A., Gentil P., Neri M., Paoli A. (2016). Effects of eight weeks of time-restricted feeding (16/8) on basal metabolism, maximal strength, body composition, inflammation, and cardiovascular risk factors in resistance-trained males. J. Transl. Med..

[53] Chen W., Cai P., Zou W., Fu Z. (2024). Psychiatric adverse events associated with GLP-1 receptor agonists: A real-world pharmacovigilance study based on the FDA Adverse Event Reporting System database. Front. Endocrinol..

[54] De Toledo F.W., Buchinger A., Burggrabe H., Hölz G., Kuhn C., Lischka E., Lischka N., Lützner A., May W., Ritzmann-Widderich M. (2013). Fasting therapy—An expert panel update of the 2002 consensus guidelines. Forsch Komplementmed..

[55] Gleason P.P., Urick B.Y., Marshall L.Z., Friedlander N., Qiu Y., Leslie R.S. (2024). Real-world persistence and adherence to glucagon-like peptide-1 receptor agonists among obese commercially insured adults without diabetes. J. Manag. Care Spec. Pharm..

[56] Gabel K., Hoddy T.W., Haggerty N., Song J., Kroeger C.M., Trepanowski J.F., Panda S., Varady K.A. (2018). Effects of 8-hour time restricted feeding on body weight and metabolic disease risk factors in obese adults: A pilot study. Nutr. Healthy Aging.

[57] Petri K.C.C., Ingwersen S.H., Flint A., Zacho J., Overgaard R.V. (2018). Semaglutide s.c. Once-Weekly in Type 2 Diabetes: A Population Pharmacokinetic Analysis. Diabetes Ther..

[58] Chavda V.P., Balar P.C., Vaghela D.A., Dodiya P. (2024). Unlocking longevity with GLP-1: A key to turn back the clock?. Maturitas.

[59] Longo V.D., Panda S. (2016). Fasting, circadian rhythms, and Time-Restricted Feeding in Healthy Lifespan. Cell Metab..

[60] Keenan S.J., Shojaee-Moradie F., Haqq A.M., Jebb S.A., Henry C.J., Noakes M., Caterson I.D., Guelfi K.J., Brinkworth G.D., Taylor P. (2022). Intermittent fasting and continuous energy restriction result in similar changes in body composition and muscle strength when combined with a 12 week resistance training program. Eur. J. Nutr..

[61] Bujdei-Tebeică I., Mihai D.A., Pantea-Stoian A.M., Ștefan S.D., Stoicescu C., Serafinceanu C. (2025). Effects of Blood-Glucose Lowering Therapies on Body Composition and Muscle Outcomes in Type 2 Diabetes: A Narrative Review. Medicina.

